# Progeria, rapamycin and normal aging: recent breakthrough

**DOI:** 10.18632/aging.100352

**Published:** 2011-07-08

**Authors:** Mikhail V. Blagosklonny

**Affiliations:** ^1^ Department of Cell Stress Biology, Roswell Park Cancer Institute, BLSC, L3-312, Elm and Carlton Streets, Buffalo, NY, 14263, USA

**Keywords:** rapamycin, aging, progeria, senescence, autophagy, obesity

## Abstract

A recent discovery that rapamycin suppresses a pro-senescent phenotype in progeric cells not only suggests a non-toxic therapy for progeria but also implies its similarity with normal aging. For one, rapamycin is also known to suppress aging of regular human cells. Here I discuss four potential scenarios, comparing progeria with both normal and accelerated aging. This reveals further indications of rapamycin both for accelerated aging in obese and for progeria.

In the last week paper in *Science Transl Med*, Francis Collins, Dimitri Krainc, Kan Cao and co-workers described that rapamycin reverses cellular phenotypes in Hutchinson-Gilford progeria syndrome (HGPS) cells [1]. Is it a co-incidence that rapamycin also suppresses senescence in regular (non-HGPS) mammalian cells [2]?

## Clearance of progerin by rapamycin

**Figure 1:**
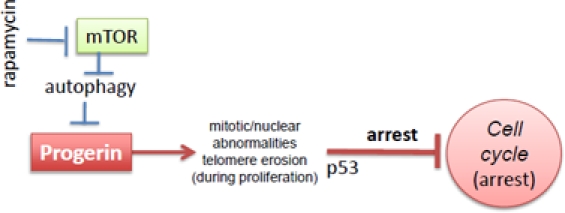
Progerin-induced replicative limit in progeric cells Rapamycin decreases levels of progerin and thus prevents telomere erosion and cell cycle arrest

Hutchinson-Gilford progeria syndrome (HGPS) is a rare genetic disorder characterized by some features reminiscent of aging, including atherosclerosis and alopecia [3-6]. The median life span is 13 years, and the main cause of death is myocardial infarction and stroke. Progeria is mainly caused by the abnormal accumulation of progerin, a mutant form of the nuclear envelope component lamin A [7, 8]. In cell culture, HGPS cells are prone to replicative senescence (Figure 1) [9-12]. Accumulation of progerin causes nuclear abnormalities, mitotic abnormalities and accelerate telomere shortening. This causes DNA damage response, p53 induction and cell cycle arrest [11, 13-16]. After a number of cell divisions in culture, cells stop proliferating (replicative senescence).

**Figure 2:**
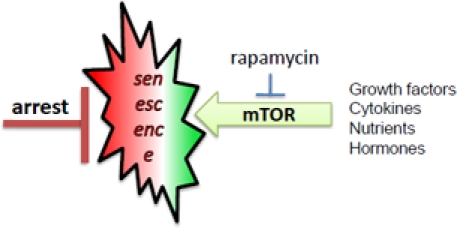
mTOR-driven senescence in arrested normal cells When cell cycle is blocked, mTOR drives senescence

Very recently Cao et al described that rapamycin stimulates clearance of progerin and therefore prevents nuclear abnormalities and delays replicative senescence [1]. Rapamycin eliminates the cause of the abnormalities and therefore is expected to be an effective treatment in progeria [1]. In addition to this strong rationale [1], there is one additional indication for rapamycin, regardless of progerin clearance, namely suppression of geroconversion (conversion from quiescence to senescence) by rapamycin [2, 17-19].

## Prevention of geroconversion by rapamycin

Normal human cells undergo replicative senescence due to telomere shortening, which causes cell cycle arrest [9, 20-22]. But cell cycle arrest is not yet senescence [23, 19]. In the young organism, post-mitotic cells are not senescent. Such cells must undergo geroconversion during lifespan. In cell culture, senescence is characterized by large flat cell morphology (hypertrophy), beta-Gal staining, hyper-secretory phenotype, activated DNA-damage response (even in the absence of DNA damage), resistance to signals (such as insulin), elevated cyclins with inappropriate drive into S-phase, and loss of proliferative potential (PP) [17, 24-42, 17]. Loss of PP, a convenient marker in cell culture, means that the cells cannot resume proliferation even when they are released from arrest, for example, by switching p21 off [2, 43]. Senescent phenotype (including loss of PP) can be linked to hyper-active growth-promoting and nutrient-sensing pathways such as mTOR (Target of Rapamycin). In proliferating cells, growth factors (and nutrients) activate cell mass growth, which is balanced by division. When the cell cycle is arrested, then activated mTOR drives the senescent morphology [2, 17]. Over-activation of the mTOR pathway causes hyper-activation and exhaustion of stem cells too [44-46].

In replicative senescence of normal human (not rodent) cells, telomere erosion during 20-70 division cycles causes DDR and cell cycle arrest. Telomere erosion is a very peculiar (and time consuming) method to achieve cell cycle arrest. One can accelerate the process. Cell cycle can be directly arrested by DNA damaging drugs, expression of p53, p21, p16 or of constantly hyper-activated Ras/Rak/Akt [47-50]. When the cell cycle is arrested but mTOR is active then cells senesce [17]. Hyper-active Ras, Raf, Akt not only can arrest cycle but additionally activate the mTOR pathway. Rapamycin and also upstream inhibitors of mTOR suppresses geroconversion in different models of premature and physiological senescence in culture [2, 17, 42, 51-55].

By causing cell cycle arrest, p53 puts such cells on the path of senescence. Simultaneously, p53 can inhibit mTOR [56-60] and can suppress geroconversion [61-66, 58]. This may determine a dual role of p53 in aging [63, 67-70].

mTOR stimulates cellular growth and functions [71-74] and cause signal (insulin) resistance by feedback loops [75-82]. Aging cells are over-activated, hyper-functional (for example, hyper-secretory) and secondary signal resistant [18]. This may result in cell malfunctions and even cellular loss (for example, loss of beta-cells in diabetes type II). Hyper-functions, malfunctions and signal-resistance in turn cause age-related diseases from metabolic syndrome, atherosclerosis and hypertension to neurodegeneration and osteroporosis [83-85]. And not coincidentally, rapamycin is indicated for prevention of all age-related diseases including cancer [83-86]. The sum of all diseases determines the risk of death [85]. And aging is defined as increase of accidence of death with age. So if TOR-dependent cellular aging increases the risk of death, then rapamycin must extend life span. Indeed, inhibition of the TOR pathway extends lifespan in span in diverse organisms from yeast to mice [87-97, 46].

## Linking progeria to aging: 4 scenarios

There are at least four models, which are not mutually exclusive

Scenario 1. Progerin is detectable in normal cells from normal elderly humans [98]. In normal human fibroblasts, telomere damage during replicative senescence activates progerin production [10]. In theory, progerin can accumulate. In this scenario, normal aging is caused by progerin or at least in some individuals accumulation of progerin is life-limiting. If so, progeria is a truly accelerated aging or at least accelerated component of aging. Still, there is no evidence so far that progerin normally reaches toxic levels. Also telomere erosion is preferentially a cell culture phenomenon. And elevated progerin production was not seen during cellular senescence that does not entail telomere shortening [10]. On the other hand, patients with dyskeratosis congenita, an inherited bone marrow failure syndrome, have very short telomeres [99]. Also, there may be synergy with additional abnormalities such as abnormalities of nuclear pore complex in aging cells [100].

Scenario 2. Normal aging is caused by overactivation of TOR-centric pathways such as mTOR, MAPK and kinases of the DNA damage response (DDR). Progerin can activate DDR [101]. In turn, DDR may activate the mTOR pathway [102]. Noteworthy, cellular senescence of regular cells is characterized by DDR even in the absence of actual DNA damage (pseudo-DDR) [103]. And pseudo-DDR is inhibited by rapamycin [103]. So there is cross talk between mTOR and DDR [104, 105]. Therefore, by activating DDR pathways, progerin might also promote geroconversion.

Scenario 3. mTOR inhibits autophagy and insufficient autophagy is involved in normal aging [106-109]. Rapamycin also causes clearance of aggregation-prone proteins [110]. In progeria, rapamycin activates clearance of progerin thus slowing down the progeric aging. Thus, rapamycin can affect both progeria and normal aging via activation of autophagy of different proteins and structures.

Scenario 4. Two different mTOR activities are responsible for deceleration of normal and progeric aging. In progeria, this is autophagy. In normal aging, this is suppression of cellular hyper-functions, such as hyperfunctions (such as secretion) and hormone-resistance. Rapamycin would be effective in both conditions but by different reasons. In analogy, rapamycin could be used for certain fungal and viral infection, even though they do not cause normal aging.

## Accelerated aging

Still, progeria is not accelerated “normal” aging exactly. What is accelerated “normal” aging or accelerated aging, for brevity. If aging is driven by inappropriate activation of nutrient-, hormone- and mitogen-sensing pathways such as mTOR, then nutrients and insulin can accelerate aging. In fact, obesity is associated with all age-related diseases and dramatically shortens life span. This is the accelerated normal aging. As an example, the maximum “years lost life” (YLL) for white men aged 20 to 30 years with a severe level of obesity (BMI >45) is 13 years, representing a 22% reduction in expected remaining life span [111]. As long ago suggested by the Russian endocrinologist Vladimir Dilman, time flies faster in the obese.

This also could be considered from the point of view of a quasi-programmed aging. Aging is not programmed of course but is an aimless continuation of a program of developmental growth [74, 112-115]. And growth is driven in part by mTOR (activated by growth factors and nutrients).

## Rapamycin for progeria and (age-related diseases)

Inhibiting farnesylation of progerin by farnesyl transferase inhibitors (FTI) prevents the nuclear blebbing of progeria and has positive effects in animal models [116-122]. Yet, the FTI lonafarnib is a relatively cytotoxic agent with gastrointestinal and hematological dose-limiting toxicities (in cancer patients) [123]. It was shown that insulin-like growth factor 1 extends longevity in a mouse model of human premature aging [124]. However, there is still a long way to clinical applications.

Therefore, rapamycin, a non-toxic prescription drug, is a very attractive option. Also, in addition to clearance of progerin, rapamycin in theory would suppress geroconversion downstream of progerin. Furthermore, rapamycin prevents atherosclerosis in animal models of accelerated atherosclerosis [125, 126] and can prevent atherosclerotic restenosis in humans [127]. And accelerated atherosclerosis is one of the main symptoms of progeria and ultimately the cause of death.

There is a misconception that rapamycin increases chances of infections and cancer. In reality, rapamycin is an effective cancer preventive agent in both animals [128, 96, 129] and humans [130, 131], in part, because it slows down organismal aging [132]. Rapamycin is not a general immunosuppressant, it induces tolerance to transplanted organs (when used in combination with immunosuppressants). “Figuratively, it transforms immunity from aged-type to infant-type” [83]. Rapamycin can actually improve responses to infections as immunostimulator [133, 46, 134]. Furthermore, to treat progeria (as well as age-related diseases in normal aging), rapamycin will be used in lower doses and intermittently, so a few (if any) side effects could be expected.

As discussed [135], an increase of lipids in blood occurs because rapamycin increases lyposysis (like starvation) and simultaneously decreases lipid entry into the tissues, including the arterial wall. Therefore, rapamycin prevents atherosclerosis in animals and humans (despite increased lipids) [125-127]. Needless to say, rapamycin is a clinically approved, non-toxic drug, which is used for many years in high and chronic doses in transplant patients. But what is about treating children? Rapamycin is successfully used for the treatment of TSC syndrome in children [136]. Now is a turn of progeria [1]. And perhaps now is the time for postponing age-related diseases of normal aging in our life time [137].
